# Supplementary material for: In-Situ Fabrication of g-C3N4/ZnO Nanocomposites for Photocatalytic Degradation of Methylene Blue: Synthesis Procedure Does Matter
Source: Nanomaterials (Basel). 2019 Feb 6;9(2):215. doi: 10.3390/nano9020215 (PMC6409917; doi:10.3390/nano9020215)
Supplement: Supplementary file 1 [file nanomaterials-09-00215-s001.pdf]

In-Situ Fabrication of g-C_3N_4/ZnO Nanocomposites for Photocatalytic Degradation of Methylene Blue: Synthesis Procedure Does Matter

Shengqiang Zhang[1], Changsheng Su[2], Hang Ren[1], Mengli Li[1,*], Longfeng Zhu[1], Shuang Ge[1], Min Wang[3], Zulei Zhang[1], Lei Li[1,*], Xuebo Cao[1,*]

[1] College of Biological, Chemical Sciences and Engineering, Jiaxing University, 118 Jiahang Road, Jiaxing 314001, China;

[2] Department of Chemical and Biomolecular Engineering, University of Notre Dame, IN 46556, USA

[3] State Key Laboratory of High Performance Ceramics and Superfine Microstructure, Shanghai Institute of Ceramics, Chinese Academy of Sciences, 1295 Ding-Xi Road, Shanghai 200050, P. R. China

* Correspondence: menglil@mail.zjxu.edu.cn (M.L. Li); leili@mail.zjxu.edu.cn (L. Li); xbcao@mail.zjxu.edu.cn (X.B. Cao); Tel.: +86-0573-8364-3852

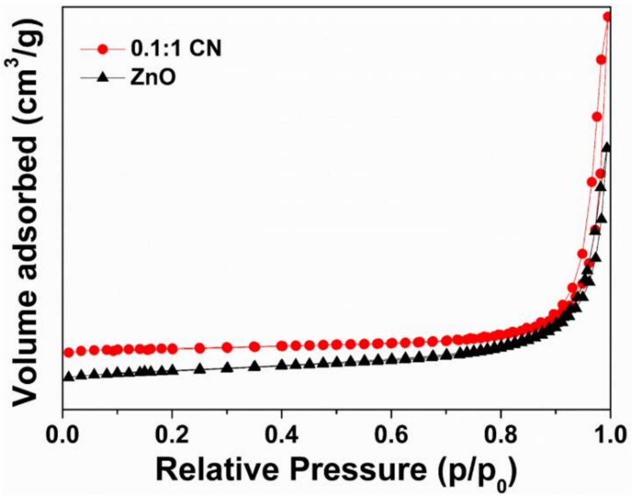

Figure S1. N_2 adsorption-desorption isotherms.

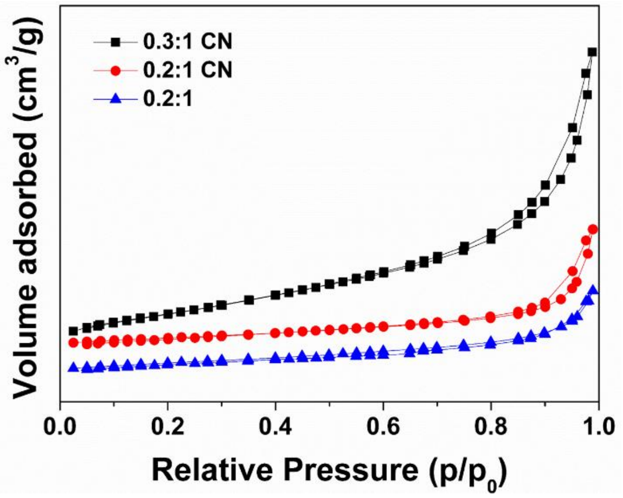

Figure S2. N_2 adsorption-desorption isotherms for three representative nanocomposites.

Nitrogen adsorption-desorption measurements were carried out at 77 K to investigate the textural and structural properties of ZnO and g-C_3N_4/ZnO (0.1:1 CN) nanocomposites. The isotherms are shown in Figure S1. Both isotherms showed a significant uptake of N_2 because of

capillary condensation in a relative pressure (P/P_0) range of 0.90-0.95. The surface area was calculated to be 11.77 $m^2 \cdot g^{-1}$ and 20.11 $m^2 \cdot g^{-1}$ for ZnO and g-C_3N_4/ZnO (0.1:1 CN) respectively. The small increase in the specific surface area in g-C_3N_4/ZnO nanocomposites was not the decisive factor for the enhancement of MB photodegradation, thus it can be inferred that the major factor depended on the enhancement of charge carrier separation efficiency.

The specific surface area of these nanocomposites became larger with the increase of g-C_3N_4 weight ratio. The surface area was enhanced to be 26.09 $m^2 \cdot g^{-1}$ and 35.15 $m^2 \cdot g^{-1}$ for 0.2:1 CN and 0.3:1 CN, respectively. However, the area was relatively smaller for x:1 samples, with the same g-C_3N_4 content as can be seen in Figure S2.

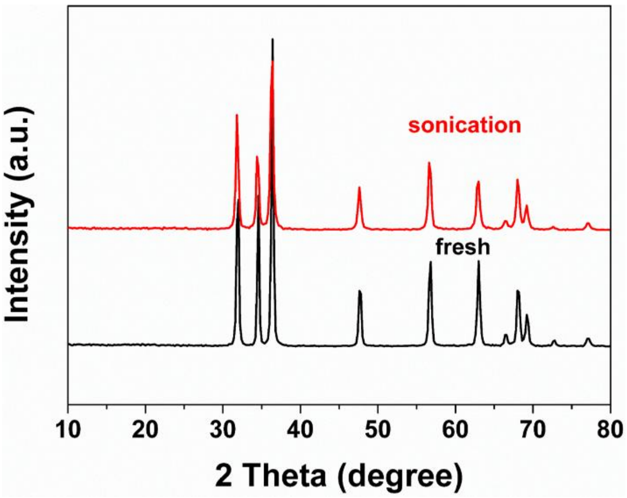

Figure S3. XRD patterns for fresh and ultrasonically treated 0.1:1 CN nanocomposite.
